# Preservative Fluid

**Published:** 1860-05

**Authors:** 


*Preservative Fluid.*—Dr. Passini recommends the following as an antiseptic mixture for the preservation of blood globules, nerves, ganglions, the retina, and the white tissues generally: Protochloride of mercury, 1 part; chloride of sodium, 2 parts; glycerine, 13 parts; and distilled water, 113 parts.—*Boston Journal.*

